# Supplementary material for: Complexome profiling of the Chlamydomonas psb28 mutant reveals TEF5 as an early PSII assembly factor
Source: Plant Cell. 2025 Mar 17;37(6):koaf055. doi: 10.1093/plcell/koaf055 (PMC12164590; doi:10.1093/plcell/koaf055)
Supplement: koaf055_Supplementary_Data [file koaf055_supplementary_data.zip › psb28_interactive.zip!/plots/intensity/qualityPlots/peptides/CAM3_Cre01.g016300.t1.1.html]

### Cre01.g016300.t1.1

Trivial name: CAM3  
SeqCoverage [median]: 30.80  
Mass: 36.17 kDa  
  
 

| Sequence | MissedCleavage | Mass [Da] | wtIntensity | muIntensity | Score |
| --- | --- | --- | --- | --- | --- |
| AQAVLKK | 1 | 756.49 | 1244.77 | 0.00 | 83.38 |
| AQVAVNTLK | 0 | 942.55 | 44438.41 | 43941.28 | 173.73 |
| ELGVEDSEQLR | 0 | 1273.62 | 0.00 | 1958.64 | 162.64 |
| ELGVEDSEQLRK | 1 | 1401.71 | 7402.63 | 9575.39 | 144.57 |
| ELSDEEYK | 0 | 1011.44 | 0.00 | 2304.87 | 111.01 |
| ELSDEEYKEALR | 1 | 1480.70 | 7161.77 | 8990.30 | 118.48 |
| ETLGFDPAR | 0 | 1004.49 | 37565.41 | 39503.05 | 163.99 |
| KAQAVLK | 1 | 756.49 | 1149.00 | 6286.69 | 99.92 |
| KLDSLLDELQK | 1 | 1300.72 | 13842.60 | 34016.70 | 173.04 |
| KLDSLLDELQKSGVDSK | 2 | 1874.00 | 2685.36 | 0.00 | 50.18 |
| LELSEVK | 0 | 816.46 | 0.00 | 3856.56 | 100.19 |
| NMSFASSQR | 0 | 1026.46 | 6763.77 | 7437.15 | 123.86 |
| STLQNLSAYLQLSHAR | 0 | 1800.95 | 9696.90 | 15053.90 | 110.35 |
| WKELGVEDSEQLR | 1 | 1587.79 | 877.96 | 1178.67 | 54.52 |
| WKELGVEDSEQLRK | 2 | 1715.88 | 1732.30 | 0.00 | 41.87 |
|  |  |  | 134560.9 | 174103.2 |  |
